# Key soil properties governing Cr(VI) retention in 16 natural soils: A comprehensive geochemical and statistical analysis

**DOI:** 10.1371/journal.pone.0338375

**Published:** 2025-12-22

**Authors:** Feng Yan, Wei Wu, Junchao Jia, Xingchang Zhang, Tongtong Wang

**Affiliations:** 1 The Chongqing Key Laboratory of Toxicology and Drug Analysis, Chongqing Police College, Chongqing, P.R.China; 2 Food, Drug, and Environmental Crime Research Institute, Chongqing Police College, Chongqing, P.R.China; 3 Bio-Agriculture Institute of Shaanxi, Shaanxi Province Academy of Sciences, Xi’an, P.R. China; 4 Institute for Interdisciplinary and Innovation Research, Xi’an University of Architecture and Technology, Xi’an, P.R. China; University of Nigeria, NIGERIA

## Abstract

Hexavalent Chromium (Cr(VI)) contamination in soils poses significant ecological risks due to its mobility and toxicity, with retention mechanisms governed by interactions between soil properties and Cr(VI). However, the quantitative roles of key soil parameters in Cr(VI) retention remain poorly resolved, particularly across diverse soil types. This study investigated Cr(VI) retention behaviors in 16 Chinese soils (15 types) through batch experiments, isothermal adsorption model, correlation analysis and path analysis. The results showed that the retention of Cr(VI) in acidic soils was significantly higher than in alkaline soils. Acidic soils (pH < 5.4) with higher concentrations of exchangeable Fe(II) (Exch-Fe(II)) exhibited strong Cr(VI) holding capabilities,while Alkaline soils (pH > 7.3) with highest content of CaCO_3_ show negligible Cr(VI) reactions.Cr(VI) retention was high at soil pH values below approximately 5.5, but declined sharply at higher pH values. The Langmuir model was only suitable for describing acidic soils (pH < 5.4), while the Freundlich equation was applicable to all soils. Correlation analysis revealed that soil pH, the content of soil organic matters(SOM), Exch-Fe(II), complexed iron (Com-Fe), and clay were significantly related to the Cr(VI) retention (*p* < 0.01), whereas the CaCO_3_ content was negatively related to the Cr(VI) retention (*p* < 0.05).Path analysis revealed that soil pH was the most important direct factor, followed by Exch-Fe(II), Com-Fe, clay, in determining Cr(VI) retention in natural soil. CEC and CaCO_3_ content had only limited directly effects on the Cr(VI) retention. Additionally, The content of SOM, Amorphous iron oxides(Amo-Fe), and Easily reducible manganese(Er-Mn) content had little directly effect on Cr(VI) retention. To validate these findings, Cr(VI) retention was measured in all soils after adjusting their pH to 4.3, 6, and 8. The results highlighted soil pH and Exch-Fe(II) content were the most decisive factors for evaluating Cr(VI) retention in natrual soils,whereas SOM content was an unreliable parameter for assessing this process.

## Introduction

Chromium (Cr), is a highly toxic heavy metal prevalent in soil ecosystems, and its environmental behavior and ecological risks have become a focal point in the field of global environmental science [1]. Cr(VI) which is nearly 1000 times more hazardous than Cr(III) and exhibits prominent bioaccumulation, genotoxicity and carcinogenicity, can enter the food chain through the soil-plant system, posing threats to human health and ecological safety [1–4]. Cr(VI) pollution stems from industrial wastewater discharge (such as electroplating,tanning and dyeing), excessive use of pesticides and fertilizers, and the mining of chromite ores [5–7]. Cr(VI) contamination in soil not only affects soil quality but also poses a potential threat to groundwater [8]. Therefore, a thorough investigation of the adsorption characteristics and mechanisms of Cr(VI) in soil is crucial for developing effective soil remediation strategies.

Upon introduction into soil systems, Cr(VI) undergoes dynamic interactions with soil components via key processes including adsorption, reduction, and precipitation, which collectively govern its environmental fate and mobility [9–11]. The intensity and pathways of these reactions were directly controlled by soil physicochemical properties, particularly key parameters such as soil pH, iron oxide (FeO_x_) content, SOM, Cation Exchange Capacity (CEC),texture and clay fraction (Clay) [11–16]. Under acidic conditions, the soil exhibits relatively strong retention capacity for Cr(VI), with a portion of this adsorption potentially involving the reduction of Cr(VI) to Cr(III) under specific redox conditions, while adsorption in alkaline soils and slightly acidic soils is significantly weakened due to deprotonation of mineral surfaces and decreaseing the adsorption and reducting of Cr(VI) [12–17]. Eary and Rai proposed that Fe(II) and SOM jointly dominate Cr(VI) reduction in acidic soils, while only Fe(II) can reduce Cr(VI) in alkaline environment [18]. Additionally, manganese oxides (MnO_x_), as the primary driver of Cr(III) oxidation, further complicate the environmental behavior of Cr(VI) [19–22]. However, existing studies were largely based on single soil types or artificial experimental systems, making it difficult to fully reflect the multifactorial synergistic effects in real soil environments.

Traditionally, correlation analysis can identify statistical associations between parameters but fails to distinguish the real contribution of a single parameter to Cr(VI) retention at complex soil system,as soils exhibits significant collinearity among different physicochemical properties [15,16,23,24]. Take SOM as an example, its correlation with Cr(VI) adsorption may only reflect its covariation with pH rather than the actual mechanism of action [16].The direct and indirect contribution of each of the characteristics with Cr(VI) retention is not clearly highlighted, and this can be achieved through path analysis. Path analysis is a statistical technique based on the analysis of structural equations and allows to provide possible causal explanations of the correlations observed between a target response variable (e.g., Cr(VI) retention capacity) and a series of explanatory variables (e.g., soil properties) [25,26].Path analysis, which partitions the total correlation into direct and indirect effects, not only helps clarify the magnitude and direction of the influence of each soil property on Cr(VI) retention but also improves the interpretability of subsequent variable selection and mechanism inference processes [25,26].

Existing studies indicated that the fate of Cr(VI) in soil (e.g., adsorption-dominated or reduction-dominated) directly determines its environmental risk, and this process is highly dependent on soil type and its intrinsic properties [11–16]. Therefore, systematically elucidating the regulatory mechanisms of key soil parameters on the Cr(VI) adsorption-reduction pathway is not only a core issue in deepening the theory of heavy metal migration but also the scientific foundation for developing precise remediation strategies.

Given the limitations of existing research, this study proposes the following scientific hypothesis: Key soil properties (such as soil pH, SOM, and Fe-Mn oxide content) significantly influence the adsorption characteristics and retention mechanisms of Cr(VI) in soil, and this influence varies significantly across different soil types. To validate this hypothesis, this study selected soil samples from 16 different regions in China and employed batch experiments, isothermal adsorption modeling, and path analysis to systematically investigate the retention mechanisms of Cr(VI) in different soils. The specific research objectives include: (1) analyzing the influence of different soil types on the retention characteristics of Cr(VI); (2) using pathway analysis to quantify the direct and indirect effects of various soil properties on Cr(VI) adsorption; (3) exploring the mechanisms by which key soil properties influence Cr(VI) retention capacity. Through this study, we aim to establish a quantitative framework linking soil key parameters to the migration and transformation of Cr(VI), thereby providing a scientific basis for developing targeted soil remediation strategies.Additionally, by elucidating the mechanisms by which key soil properties influence Cr(VI) adsorption, this study offers theoretical support for developing more effective soil remediation strategies, thereby reducing the potential threats posed by Cr(VI) to the ecological environment and human health.

## Materials and methods

### Soil sampling and preparation

**Fig 1:**
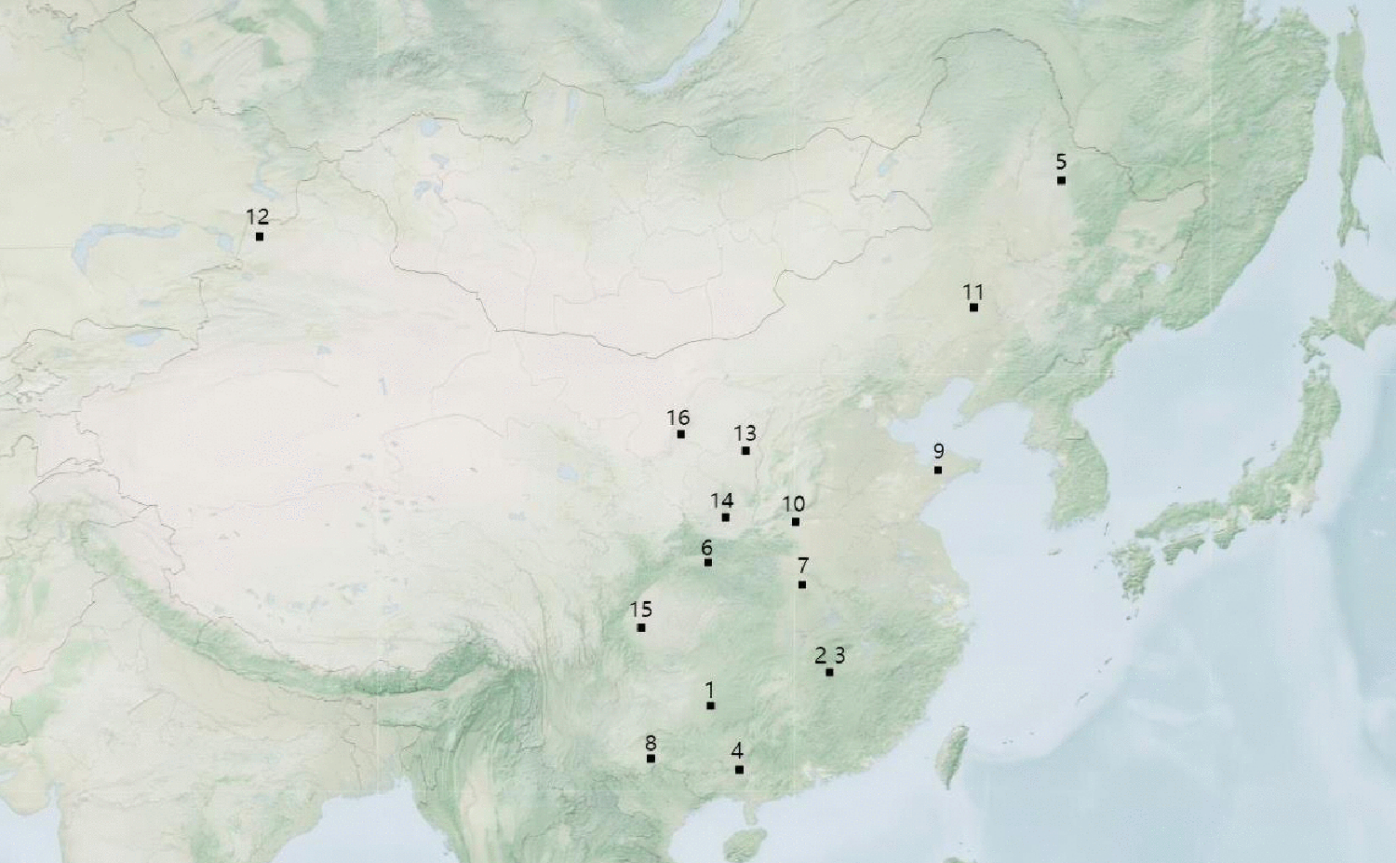
Soil sampling sites.

The sampling sites were shown in Fig 1. At each site, 5.0 kg of surface soil (0–20 cm depth) was collected in plastic bags using a multipoint sampling method. All sampling sites were located in agricultural land. The collected soils were transported to the laboratory by train under airtight conditions and at ambient temperature. A total of 16 soil samples were obtained from different regions of China, covering a wide range of soil properties and representing the major soil types in the country.

Based on the Chinese Soil Classification System (S1 File), the classification of the 16 samples (which belong to 15 soil types) were categorized as follows::Soil 1-Yellow soil; Soil 2-Red soil (paddy field); Soil 3-Red soil (upload field); Soil 4-Lateritic Red Soil; Soil 5-Black Soil; Soil 6-Yellow-brown soil; Soil 7-Yellow-Cinnamon Soil;Soil 8-Paddy Soil; Soil 9-Fluvo-Aquic Soil; Soil 10-Cinnamon Soil; Soil 11-Castanozems; Soil 12-Chernozem; Soil 13-Cultivated lossial Soil; 14-Lou Soil; Soil 15-Purplish Soil; Soil 16- Cumulated irrigated Soil.

Soil Sample Preparation: Collected soil samples were first air-dried naturally in a well-ventilated laboratory (protected from direct sunlight to prevent mineral alteration) on clean polyethylene trays, with gentle turning every 2–3 days for uniform moisture evaporation. After about one month of drying (confirmed by consistent dryness), the soils were ground in an agate mortar until fully pulverized. The powder was then sieved through a 0.147 mm nylon mesh using a mechanical shaker for 10 minutes to obtain a homogeneous fine fraction.

Storage and Experimental Setup: The processed samples were stored in plastic containers under controlled low-humidity conditions (RH < 40%), then utilized for soil physicochemical analyses and batch experiments.

### Soils characterization analysis

PH Measurement:Soil samples with deionized water (solid-liquid mass ratio 1:1), were shaked at 180 rpm for 1 hour,and then allowed to stand overnight at 25 ± 1°C to equilibrate, a pH meter (PHS-3C model, Inesa scientific instrument Co., Ltd., China) was used to determine the pH of the soil solution.

SOM Measurement:SOM was determined via the modified Walkley-Black method (K_2_Cr_2_O_7_-H_2_SO_4_ oxidation), which oxidizes organic carbon (SOC) and converts results to SOM using a stoichiometric coefficient (SOM = SOC × 1.724) [27].

Soil Clay Content Measurement: Soil was treated with H_2_O_2_ (H_2_O_2_:water = 1:4) to remove OM, followed by particle size distribution analysis using a Laser particle size analyzer (Malvern MASTERSIZER 3000 model, UK), and data processing [27].

Soil Exch-Fe(II) Concentration Measurement:Soils were extracted with 0.1 mol/L Al_2_(SO_4_)_3_, and detected Fe(II) in extracts by the spectrometer using the phenanthroline method [27].

Soil Amo-Fe content Measurement: Soils were extracted by 0.2 mol/L (NH_4_)_2_C_2_O_4_, and detected total iron in extracts by Atomic Absorption Spectroscopy (AAS, iCE 3500 model, Thermo Fisher Scientific, USA) [27].

Soil Com-Fe content Measurement: the soils were extracted by 0.1 mol/L,Na_4_P_2_O_7_ (pH = 8.5), and detected iron in extracts by AAS [27].

Soil Er-Mn Content Measurement: soil was extracted by 1.0 mol/L,MgSO4 and 1.0 mol/L MgSO_4_–0.2 M hydroquinone (1:1) respectively, measured the total manganese by AAS, the concentration of Er-Mn were the manganese difference between these two extracts [27].

Soil CaCO_3_ content: It was measured by the gasometric method by measuring CO_2_ gas released from the reaction of CaCO_3_ with excess hydrochloric acid (HCl) (S2 File) [27].

Cation Exchange Capacity (CEC) Measurement: CEC was determined by the method of ammonium acetate exchange (S2 File) [27].

All chemical reagents used in this study are of analytical grade and do not require further purification. They were purchased from Sinopharm Chemical Group Co., Ltd.

### Batch experiments

Due to the complex nature of Cr(VI) reactions with soil components, it is difficult to distinguish the above mechanism of Cr(VI) retention in real soil systems. Hence, the collective term “Cr(VI) retention”is used to quantify the overall removal of Cr(VI) by all the reactions mentioned above. In this study, the total Cr(VI) loss in the batch experiment was accounted for Cr(VI) retention.

The investigation of Cr(VI) retention isotherm was conducted by batch equilibrium technique. Stabilized pH which was close to the natural soil pH by shaking soil-deionized water for 8 h and standing the solution the whole night before adding K_2_Cr_2_O_7_ to start batch experiment. Another advantage of this method is that it can decrease the rate limitation by soil ingredients dissolution. The specific steps were as follows:27mL deionized water (0.01 mol/L KNO_3_ as background electrolyte) was added to each polyethylene tube which contained 3.000 ± 0.003 g of each soil, Samples shaken 8 h and stood the solution the whole night at 25 ± 1 °C, at next day, adding 3 mL different K_2_Cr_2_O_7_ concentrations (0.01M KNO_3_ as background electrolyte), then shake with 180 rpm at 25 ± 1 °C continuously for 48 h, filtered and determinate Cr(VI) by UV-vis spectrophotometer (UV-2600 model, Shimadzu Corporation, Japan). Filtrate was placed in a 50 ml colorimetric tube and dilute with water to the mark. 0.5 ml Mixed sulfuric acid solution (H_2_SO_4_:H_2_O=Volume ratio1:1) and 0.5m1 phosphoric acid solution (H_3_PO_4_:H_2_O=Volume ratio 1:1) were added and well shaked,and then 2 ml of color reagent (C_13_H_14_N_4_O 0.2g, was dissolved in 50 ml of acetone then diluted with water to 100 ml) were added and shake well, After 5–10 minutes, The sample was detected at wavelength of 540 nm by UV-vis spectrophotometer for Cr(VI) concentration.

### Model analysis

The observed change in Cr(VI) concentration in the aqueous phase was considered to be due solely to Cr(VI) retention in each soil. And determinations were conducted in triplicate. After checking the slurry pH from the initial to batch experiments finish, the solution pH did not undergo a significant change over the experiments period, changing within±0.05.During all experiments, deionized water was used.

The adsorption capacity (*Q*_e_, mg/g) at equilibrium was calculated as follows:


$$Q_{e}=(C_{0}-C_{e})\frac{V}{m}$$

Where *C*_0_ and *C*_*e*_ are the initial and equilibrium concentrations (mg/L), respectively. *m* is the mass of added the soil samples (g), and *V* is the solution volume (L).

To better investigate the retention characteristics, two isotherms models (Langmuir and Freundlich models) were used. Cr(VI) retention isotherm data were fitted to Langmuir’s model (Eq 1) and Freundlich’s model (Eq 2).

The Langmuir isotherm (Eq 1) assumes a monolayer sorption process, defined by a fixed number of uniform sorption sites and no adsorbate migration in the surface plane, where the adsorbate is incrementally adsorbed onto the adsorbent’s uniform surface until a steady value reflecting the maximum adsorption capacity is reached [28–30]. The model is expressed as in Eq 1 [28–30]:


$$\text{Langmuir}\;\text{model}:\;\;\frac{C_{e}}{Q_{e}}=\frac{\text{1}}{Q_{m}K_{L}}+\frac{C_{e}}{Q_{m}}$$ (1)

Where *C*_*e*_ is the concentration of Cr(VI) in the solution at adsorption equilibrium (mg/L), *Q*_*e*_ is the retention amount of Cr(VI) at adsorption equilibrium (mg/g). Where *K*_L_ (the Langmuir adsorption coefficient (mL/g)) is related to the free energy of adsorption, *Q*_*m*_ is the theoretical maximum adsorption capacity (mg/g).

The Freundlich model (Eq 2) assumes a non-uniform, more complex adsorption surface characterized by multi-layered interactions, resulting in unevenly distributed adsorption heat, and it can simulate the adsorption behavior of highly heterogeneous adsorbents with the adsorption heat at the adsorption centers decreasing exponentially [28–30]. The equation expressions are as in Eq 2:


$$\text{Freundlich}\;\text{model}:\;\text{log}(Q_{e})=\text{log}(K_{F})+\frac{\text{1}}{n}\text{log}(C_{e})$$ (2)

Where *K*_F_ (the distribution coefficient) refers to the adsorption capacity of the adsorbent and 1/*n* (correction factor) is the heterogeneity of the surface.

### Retention experiments of Cr(VI) under different soil pH systems

Three different pH buffers were prepared separately: acetic acid-sodium acetate buffer (0.2 mol/L, pH ≈ 4.0), 2-(N-morpholino)ethyl sulfonic acid buffer (1 mol/L, pH ≈ 6.0), and 4-hydroxyethyl piperazine ethyl sulfonic acid buffer (1 mol/L, pH ≈ 8.0). Adjust the pH of each buffer solution using 0.5–1.0 mol/L NaOH. Weigh 3.000 ± 0.003 g of 16 soil samples into 50 mL centrifuge tubes, with a total of 16 × 3 × 3 test samples (16 soil types, 3 pH buffer solutions, repeated 3 times), and then add 29 mL of pH buffer solution, and 1 mL of K_2_Cr_2_O_7_ (containing Cr(VI) at a concentration of 600 mg/L) to achieve an initial Cr(VI) concentration of 20 mg/L. The tubes were then placed in a shaker and shaken continuously with 180 rpm at 25 ± 1 °C for 48 h. After shaking, the samples were taken and filtered. Finally, the Cr(VI) concentration in the filtrate was measured using the same method as above. The difference between the initial Cr(VI) concentration in the solution and the Cr(VI) concentration in the filtrate after equilibrium represents the total adsorption of Cr(VI) in the soil.

### Data process

This involved cluster analysis and principal component analysis (PCA) relevant models. A detailed description of the analytical models used for this work can be found on this cloud computing platform. Data analysis was performed using SPSS 22.0, with one-way analysis of variance (ANOVA) and least significant difference (LSD) multiple comparisons (*p* < 0.05). Additionally, OriginPro 2021 software was used for model parameter fitting and plotting. The correlation analysis and path analysis were conducted using OriginPro 2021 software (OriginLab Corporation, USA) to elucidate the relationships between key variables and quantify their direct and indirect effects.

## Result and discussion

### Soil basic physical and chemical properties analysis

The physicochemical properties of the 16 tested soils exhibited substantial variability, reflecting diverse soil types and geochemical conditions (Table 1). Soil pH ranged from acidic (5.04, Soil 1) to strongly alkaline (8.25, Soil 16), with a clear demarcation between acidic (pH < 7.0, Soils 1–7) and alkaline soils (pH > 7.0, Soils 8–16). Acidic soils generally displayed higher SOM content (11.85–36.03 g/kg) compared to alkaline soils (4.80–37.18 g/kg), though Soil 12 (pH 7.68) showed an anomalous SOM value of 37.18 g/kg. Clay content varied widely across samples (16.41–53.25%), with acidic soils (Soils 1–4) containing higher clay fractions (41.00–53.25%) than most alkaline soils (16.41–47.41%). Notably, Amo-Fe content remained relatively stable (1.25–3.16 g/kg), while Com-Fe exhibited significant variation (1.7–1182.4 mg/kg), with acidic soils (Soils 1–4) demonstrating elevated Com-Fe levels (547.6–1182.4 mg/kg). Er-Mn showed extreme variability (6.24–254.51 mg/kg), particularly in Soil 5 (218.79 mg/kg) and Soil 6 (254.51 mg/kg), suggesting localized Mn enrichment. CEC ranged from 12.16 to 49.35 c mol^+^/kg, with higher values observed in acidic soils (e.g., Soil 5: 49.35 c mol^+^/kg). Calcium carbonate (CaCO_3_) content increased markedly in alkaline soils (4.924–114.055 g/kg), peaking in Soil 16 (114.055 g/kg), whereas acidic soils maintained lower levels (4.924–11.919 g/kg). Exchangeable Fe(II) was predominantly detected in acidic soils (33.45–64.44 mg/kg), with Soil 8 (pH 7.55) being an exception (64.44 mg/kg), while most alkaline soils lacked measurable Fe(II). These trends highlight distinct geochemical regimes: Acidic soils were characterized by higher SOM, clay, and Fe(II) availability, whereas alkaline soils featured elevated CaCO_3_ and reduced Fe(II) activity. The pronounced heterogeneity in soil properties, particularly soil pH, Fe(II), and CaCO_3_, establishes a robust foundation for investigating their roles in Cr(VI) adsorption-reduction dynamics. Such variability ensures the representativeness of the dataset in capturing soil-specific responses to Cr(VI) retention, aligning with subsequent mechanistic analyses.

**Table 1:** Basic physical and chemical properties of the test soils.

Soil number	pH	SOM (g/kg)	Clay (%, < 0.01mm)	Amo-Fe (g/kg)	Com- Fe (mg/kg)	Er-Mn (mg/kg)	CEC (c mol^ + ^/kg)	CaCO_3_ (g/kg)	Exch-Fe(II) (mg/kg)
1	5.04	36.03	53.16	1.83	1182.4	36.70	26.99	6.757	56.26
2	5.28	33.93	48.08	2.45	547.6	21.17	25.85	11.919	38.13
3	5.34	33.68	53.25	3.16	905.3	48.51	31.09	10.806	33.45
4	5.38	18.10	41.00	1.74	685.0	6.24	18.32	9.456	36.67
5	5.72	31.78	37.48	2.30	586.8	218.79	49.35	4.924	--
6	5.80	11.85	39.90	2.57	266.1	254.51	36.11	6.457	--
7	6.14	10.93	36.95	2.39	139.6	190.16	40.45	9.253	--
8	7.55	11.75	46.57	2.88	43.6	46.93	29.50	7.733	64.44
9	7.27	10.97	28.33	1.92	98.2	141.70	36.57	8.753	--
10	7.60	9.33	30.96	2.63	34.5	171.36	34.97	9.127	--
11	7.44	13.62	16.41	1.25	69.8	80.98	17.18	16.959	--
12	7.68	37.18	19.72	1.61	91.6	185.81	34.74	7.026	--
13	7.37	4.80	19.79	1.50	1.7	67.74	12.16	81.373	--
14	7.75	9.53	35.10	2.01	25.4	151.03	23.34	54.997	--
15	7.84	5.69	35.22	1.66	15.3	170.98	22.42	41.841	--
16	8.25	8.80	47.41	1.62	264.4	43.62	26.53	114.055	--

Note: “--” indicates not detected, Cr(VI) content at these soils were <1.0 mg/kg.

### Cr(VI) retention in 16 soil samples

**Fig 2:**
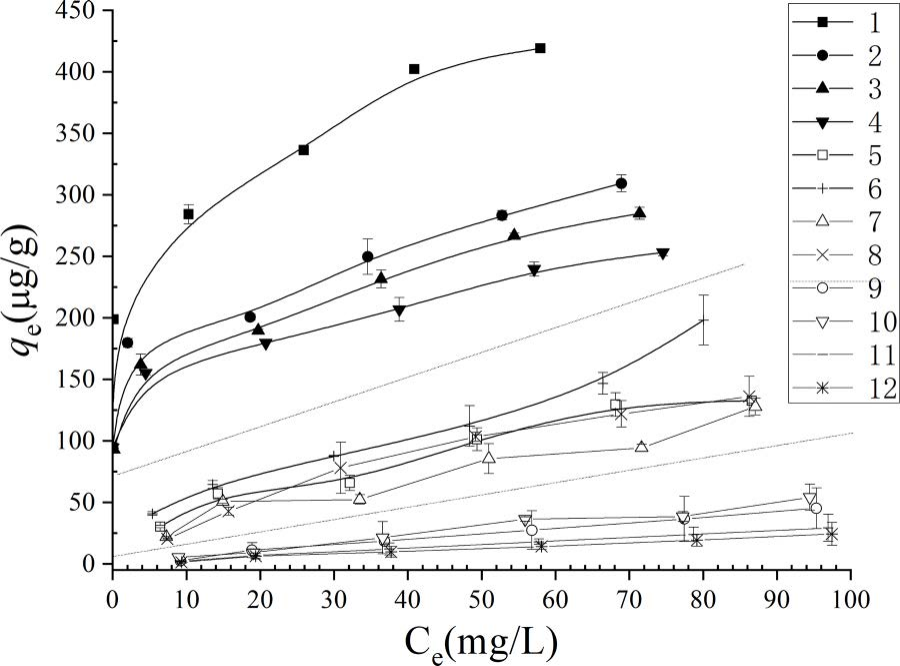
Isotherms of Cr(VI) retention in different soil samples.

A significant variation in Cr(VI) retention capacities was observed among the tested soils (Fig 2). The *Q*_100_ values, defined as Cr(VI) retention at an initial concentration of 100 mg/L, ranged from 0 to 419.192 μg/g. The retention of Cr(VI) was strongly pH-dependent, as evidenced by the fact that all soils exhibiting non-Cr(VI) retention mechanisms were alkaline in nature. Cr(VI) adsorption isotherms for 12 natural soils were presented in Fig 2 (soils12−16 showed negligible Cr(VI) retention). The Freundlich equation (Eq 2) generally provided an superior description of Cr(VI) retention across all soils (Table 2, fitted R^2^ values: 0.823–0.985), due to more complex reactions such as electrostatic adsorption,coordination complexation and reduction [31]. Of particular interest, only acidic soils with highest Exch-Fe(II) (soils 1, 2, 3, and 4) conformed well to the Langmuir model (Eq 1 R2=0.976–0.986, *p* < 0.01), suggesting barrier-free reaction (mostly large amounts of reduction) [17,31]. *Q*_100_ values (Cr(VI) retention at 100 mg/L initial concentration, Table 2) were employed to facilitate a comparative assessment of Cr(VI) retention across different soil types.

**Table 2:** Results of Langmuir and Freundlich model fittings for Cr(VI) retention on Soils.

Soil number	Langmuir	*K*_L_	*Q*_m_	Freundlich	*n*	*Q*_100_
1	y = 2.3775x + 5.3912 R^2 ^= 0.9861^**^	0.4410	420.61	y = 0.1141x-0.6095 R^2^ = 0.9212^**^	8.76	419.19
2	y = 3.2361x + 13.195 R^2 ^= 0.9762^**^	0.2453	309.01	y = 0.1475x-0.8177 R^2 ^= 0.8232^*^	6.78	309.33
3	y = 3.4499x + 16.567 R^2^ = 0.9791^**^	0.2082	289.86	y = 0.1765x-0.8998 R^2^ = 0.9757^**^	5.67	284.90
4	y = 3.9065x + 16.702 R^2^ = 0.9827^**^	0.2339	255.98	y = 0.1735x-0.9405 R^2^ = 0.9209^**^	5.76	253.21
5	y = 5.1513x + 215.53 R^2^ = 0.8867^**^	0.0239	194.13	y = 0.5613x-1.953 R^2^ = 0.9625^**^	1.78	132.69
6	y = 3.8794x + 171.27 R^2^ = 0.7787^*^	0.0227	257.77	y = 0.5387x-1.8065 R^2^ = 0.9623^**^	1.86	198.12
7	y = 5.2952x + 315.68 R^2^ = 0.7550^*^	0.0167	188.85	y = 0.6222x-2.1429 R^2^ = 0.9258^**^	1.61	127.87
8	y = 3.3261x + 331.56 R^2 ^= 0.9716^**^	0.0100	300.65	y = 0.7929x-2.3564 R^2^ = 0.9842^**^	1.26	136.14
9	y = −7.6369x + 2629.1 R^2 = ^0.17	–	–	y = 1.1454x-3.5675 R^2^ = 0.9575^**^	0.87	45.23
10	y = 0.1541x + 1776.1 R^2^ = 0.0006	–	–	y = 0.9945x-3.2404 R^2^ = 0.9845^**^	1.00	54.18
11	y = −4.7826x + 3591.2 R^2 = ^0.093	–	–	y = 1.0767x-3.6423 R^2^ = 0.982^**^	0.93	29.30
12	y = −13.364x + 4989.7 R^2^ = 0.134	–	–	y = 1.1324x-3.8318 R^2^ = 0.952^**^	0.88	24.31

Note: “-” indicates that the fit is not convergent. “Q_100_” denotes the retention capacity of different soil samples under the the initial Cr(VI) concentration is 100 mg/L.“*” and “**” indicate significant (*p* < 0.05) and highly significant (*p* < 0.01) levels. Soils 13–16 were not listed as having negligible Cr(VI) retention.

#### Soils were categorized into four distinct groups based on their retention characteristics.

Group 1 (Soils 1−4) with a soil pH ranging from 5.04 to 5.38, and highest Ex-F(II),exhibited the highest Cr(VI) retention capacities (*Q*_100_ = 253−419 μg/g,), demonstrated superior fit to the Langmuir model (R^2^ = 0.976–0.986) compared to Freundlich model, and showed significantly higher *K*_L_ and n values than other soils (Table 2). These results suggest that soils in Group 1 possess a strong Cr(VI) retention capacity. The exceptional retention capacity in this group can be attributed to their acidic environment which facilitates direct reductive interactions between Cr(VI) and reductants such as SOM and Exch-F(II) [13,17].

Group 2 (Soils 5–8) exhibited intermediate Cr(VI) retention capacities (*Q*_100_ ranged from 127–198 μg/g). These soils were best described by the Freundlich model compared to the Langmuir model, with *n* values ranging from 1.26 to 1.78 (Table 2), indicating relatively low Cr(VI) adsorption affinity [29]. The soils, with pH values varying between 5.72 and 6.14 (except soil 8, pH = 7.55). Soil 8, as the only alkaline soil with exceptional Cr(VI) retention (*Q*_100_ = 136.14 μg/g) at pH 7.55, may be due to its higher extractable Ex-Fe(II) content (Table 1), which facilitates Cr(VI) reduction through Fe(II)-mediated reactions, particularly under alkaline conditions where Fe(II)/ Fe minerals become primary reductants [18].

Group 3 (Soils 9–16) with neutral pH from 7.27 to 7.60 exhibited minimal Cr(VI) retention capacities (*Q*_100_ = 24–45 μg/g). These soils demonstrated weak Cr(VI) retention affinity as evidence by excellent Freundlich equation description (*R*^2^ = 0.912–0.948) with n values approaching 1 (0.87–1.26) (Table 2), indicating low Cr(VI) retention capacity and near-ideal adsorption behavior at low concentrations [29,32].

Group 4 (Soils 13–16) exhibited negligible Cr(VI) retention capacity (*Q*_100_ = 0 μg/g) within 2 days. These soils could not be adequately modeled by either the Freundlich or Langmuir models due to their extremely trace Cr(VI) adsorption potential. The soils in this group exhibited no reactivity toward Cr(VI). Soils in Group 4 exhibited the highest pH values (7.37–8.25), notably elevated CaCO_3_ concentrations compared to other groups (Table 1).

Thus the natural soil may be roughly divided roughly into 4 groups for Cr(VI) retention: (1) Large Cr(VI) retention soil (soil pH may be less than 5.5); (2)Mediate Cr(VI) retention soil (soil pH may from 5.5–6.5); (3)Slight Cr(VI) retention soil (soil pH ≈ 7–8); (4) Negligible Cr(VI) retention soil (soil pH may more than 7.5 with higher content of CaCO_3_). Furthermore, soil types play an unclear role in Cr(VI) retention with only the trend was that Alfisols (such as Yellow Soil,Red Soil,Lateritic Red Soil) in southern China had more Cr(VI) retention due to lower pH and higher content of FeO_x_.The observed soil type variations were largely explained by soil properties (soil pH, Exch-Fe(II), and CaCO_3_) rather than taxonomic classification [14–16].

### The correlation of soil properties with Cr(VI) retention

The correlation was used to understand the impact of soil properties on Cr(VI) retention (*Q*_100_). Specifically, correlation analysis (Table 3) was conducted to establish relationships between soil properties and Cr(VI) retention. Soil pH exhibited the strongest negative correlation with *Q*_100_ (*r* = −0.9108^**^, *p* < 0.01), confirming its dominant role in controlling Cr(VI) behavior through both electrostatic interactions and redox potential modulation. Com-Fe content demonstrated the second strongest positive correlation (*r* = 0.8856^**^, *p* < 0.01), highlighting its dual function as adsorption sites and electron donors for Cr(VI) reduction. Exch-Fe(II) showed a significant positive correlation (*r* = 0.7487^**^, *p* < 0.01), directly supporting its role as the primary reductant. Clay content displayed a moderate positive correlation (*r* = 0.7146^**^, *p* < 0.01), suggesting its importance in physical adsorption through surface area and charge characteristics. SOM’s positive correlation (*r* = 0.6515^**^, *p* < 0.01) appeared mediated through complexation with Fe oxides rather than direct interaction, as evidenced by its stronger correlation with Com-Fe (*r* = 0.7242^**^) than with *Q*_100_. The negative correlation of CaCO_3_ (*r* = −0.5169^*^, *p* < 0.05) reflects its pH-buffering capacity that inhibits reduction processes while potentially competing with chromate for adsorption sites through carbonate ion exchange.

**Table 3:** Correlation coefficients of soil properties with Cr(VI) retention.

Soil Properties	pH	SOM	Exch-Fe(II)	Amo-Fe	Com- Fe	Er- Mn	CEC	Clay	CaCO_3_	*Q*_100_
pH	1.0000									
SOM	−0.6226^**^	1.0000								
Exch-Fe(II)	−0.4884	0.4239	1.0000							
Amo-Fe	−0.3922	0.2009	0.3796	1.0000						
Com- Fe	−0.8337^**^	0.7242^**^	0.5946^*^	0.2312	1.0000					
Er- Mn	0.1794	−0.1682	−0.6791^**^	0.1024	−0.4188	1.0000				
CEC	−0.2192	0.3104	−0.1658	0.5171^*^	0.1001	0.6346^**^	1.0000			
Clay	−0.5456^*^	0.3350	0.6694^**^	0.5795^*^	0.6893^**^	−0.3804	0.1742	1.0000		
CaCO_3_	0.5504^*^	−0.4855	−0.3341	−0.4413	−0.2979	−0.2293	−0.4784	−0.0678	1.0000	
*Q*_100_	−0.9108^**^	0.6515^**^	0.7487^**^	0.4289	0.8856^**^	−0.3910	0.1049	0.7146^**^	−0.5169^*^	1.0000

Note: “* and ** indicate significant differences at the 0.05 and 0.01 levels, respectively, in variance analysis, corresponding to p-values reaching significant and extremely significant levels.”

Interparameter correlations exposed intricate indirect effects on Cr(VI) retention dynamics. The strong negative association between pH and Com-Fe (*r* = −0.8337^**^) suggests acidic conditions favor Fe oxide complexation, creating synergistic enhancement of Cr(VI) retention. Exch-Fe(II)‘s dual correlation with clay (*r* = 0.6694^**^) and Com-Fe (*r* = 0.5946^*^) indicates its spatial co-distribution with these components, potentially forming microsites for coupled adsorption-reduction processes. The unexpectedly weak correlation between CEC and *Q*_100_ (*r* = 0.1049) contrasts with conventional adsorption theories, possibly due to chromate’s divalent anion characteristics being less influenced by cation exchange sites. Er-Mn’s negative correlation with Exch-Fe(II) (*r* = −0.6791^**^) suggests competitive redox relationships, though its direct impact on *Q*_100_ remains insignificant (*r* = −0.3910). These findings were consistent with previous studies by Rassaei et al. [14], further reinforcing the general understanding of the influence of soil properties on Cr(VI) retention. However, it is worth noting that CEC exhibited different behavior in this study, and its specific impact on Cr(VI) retention dynamics requires further investigation. Overall, the correlation analysis provides a comprehensive overview of the relationship between soil properties and Cr(VI) retention, highlighting the key factors that significantly influence this process.

### Key soil properties affecting Cr(VI) retention by path analysis

Standard correlation analysis alone proved insufficient for conclusively determining the relative importance of individual soil properties due to significant multicollinearity among variables. As soil pH, SOM content, clay percentage, CaCO_3_ content, and exchangeable Fe(II) all exhibited positive correlations with *Q*_100_ (*p* < 0.05), these factors were highly intercorrelated (Table 3).This creates a methodological challenge where the true influence of individual variables may be obscured or exaggerated through their mutual associations [33].Path analysis, was conduct to identify the direct effect of an independent variable on Cr(VI) retention (Table 4), thereby overcoming the inherent limitations of simple bivariate correlations analysis [25,26].

**Table 4:** Direct and indirect path coefficients between soil properties and Cr(VI) retention.

Soil Properties	pH	SOM	Exch-Fe(II)	Amo-Fe	Com-Fe	Er-Mn	CEC	Clay	CaCO_3_	Correlation Coefficient
pH	**−0.4867** ^**^	−0.0294	−0.1135^*^	0.0130	−0.1276^*^	0.0110	0.0274	0.1236^*^	−0.0815	−0.9108^**^
SOM	0.3030^**^	**0.0472**	0.0985	−0.0067	0.1109^*^	−0.0103	−0.0388	0.0759	0.0719	0.6515^**^
Exch-Fe(II)	0.2377^*^	0.0200	**0.2324**	−0.0126	0.0910	−0.0416	0.0207	0.1516	0.0495	0.7487^**^
Amo-Fe	0.1909^*^	0.0095	0.0882	**−0.0332**	0.0354	0.0063	−0.0647	0.1312	0.0653	0.4289
Com-Fe	0.4058^**^	0.0342	0.1382	−0.0077	**0.1531** ^*^	−0.0256	−0.0125	0.1561^*^	0.0441	0.8856^**^
Er-Mn	−0.0873	−0.0079	−0.1578 ^*^	−0.0034	−0.0641	**0.0612**	−0.0794	−0.0861	0.0339	−0.3910
CEC	0.1067^*^	0.0146	−0.0385	−0.0172	0.0153	0.0388	**−0.1251** ^*^	0.0394	0.0708	0.1049
Clay	0.2655^*^	0.0158	0.1556 ^*^	−0.0193	0.1055^*^	−0.0233	−0.0218	**0.2265** ^*^	0.0100	0.7146^**^
CaCO_3_	−0.2678^*^	−0.0229	−0.0777	0.0147	−0.0456	−0.0140	0.0598	−0.0153	**−0.1480** ^*^	−0.5169^*^

Note: “^*^” and “^**^” indicate significant differences of Correlation coefficient at the 0.05 and 0.01 levels, respectively, in the analysis of variance, i.e., the p-values reached the levels of significant difference or extremely significant difference, respectively. The numbers at diagonal were the direct coefficient of a factor in influencing Cr(VI) retention; the other numbers at row were a factor’s indirect coefficient via other factors. The sum of a factor’s direct coefficient and indirect coefficients at a row was equal to the factor’s correlation coefficient with Cr(VI) retention.Absolute value of the path coefficient can reflect the “effect strength”.For most studies in the field of soil or environment, an absolute value of 0.1–0.3 is “meaningful“ (“*”), and>0.3 is “sigifigant” meaningful (“**”).

### Effect of soil pH

**Fig 3:**
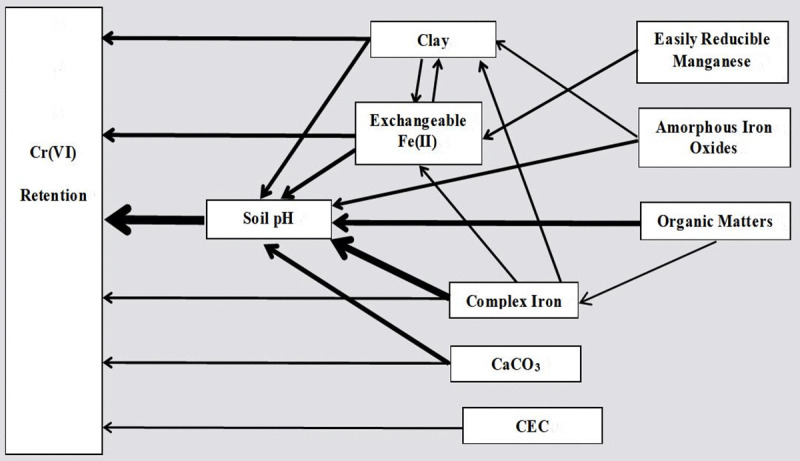
Direct and indirect effects of soil properties on Cr(VI) retention based on path analysis.

Path analysis revealed soil pH as the dominant regulator of Cr(VI) retention, exhibiting the highest direct effect coefficient (−0.4867, Table 4). Other properties demonstrated substantially smaller direct effects compared to their pH-mediated indirect influences. Com-Fe, SOM, clay minerals, CaCO_3_, Amo-Fe, and Exch-Fe(II) exhibit stronger pH dependency compared to other soil properties, as evidenced by their larger indirect effect coefficients (ranging from 0.2377 to 0.4058) driven by pH (Table 4, first column).This study further elucidates the critical role of pH in governing Cr(VI) mobility by influencing soil properties interact with Cr(VI) (Fig 3). The mechanisms maybe: (1) altering Cr(VI) speciation (e.g., HCrO₄ ⁻ /Cr₂O₇²⁻); (2) modulating the surface charge (positive or negative) of soil particles (e.g.,SOM,clay minerals), which affects adsorption; (3) regulating the soil redox environment (e.g.,SOM,clay,Com-Fe,); and (4) promoting or inhibiting the dissolution/precipitation of soil reactants (e.g.,Fe(II)/Fe(III) from clay minarals) [12–14,16,18,34–36].

**Fig 4:**
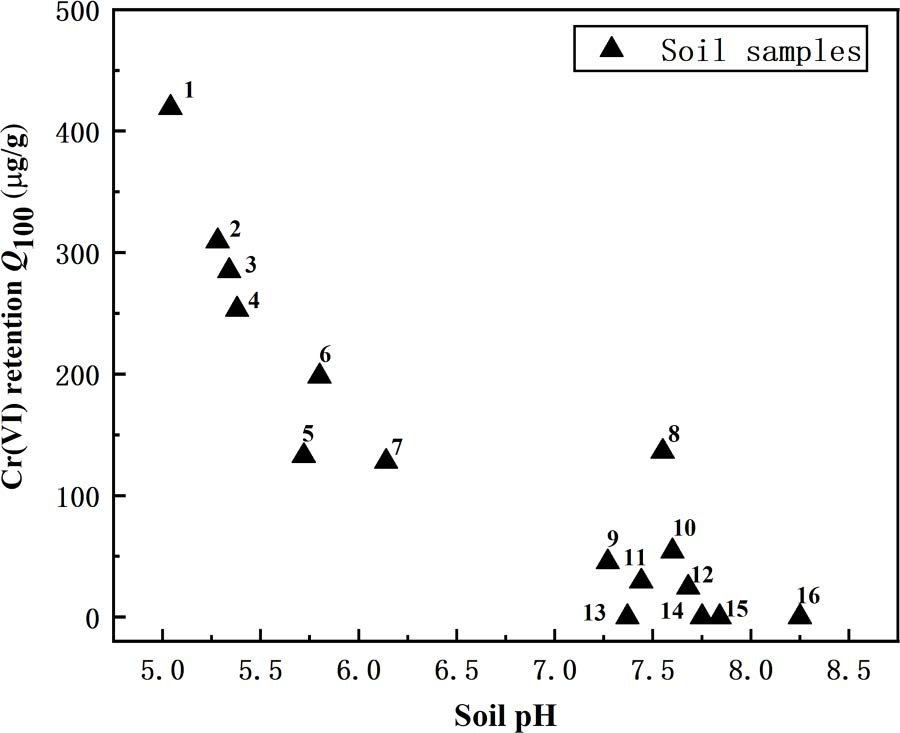
Cr(VI) retention vs. soil pH in different soil samples.

Fig 4 corroborates this finding, showing a significant negative relationship between soil pH and Cr(VI) retention across diverse soil matrices. Retention capacity decreased markedly with increasing pH, regardless of variations in other properties.Soil pH is the controlling factor, and a pH of approximately 5.5 may represent a critical inflection point. This is because pH < 5.5 promotes Cr(VI) adsorption and reduction, whereas higher pH values suppress these process.These findings align with previous studies emphasizing pH’s primacy over other soil parameters in controlling Cr(VI) retention in soils [16,36].

### Effect of Exch-Fe(II) content and clay percentage

Exchangeable Fe(II) and clay content emerged as secondary controlling factors (Fig 3). The direct (0.2324) and pH-mediated indirect (0.2377) coefficients for Fe(II) (Table 4) indicate dual mechanisms: direct chemical reduction and pH-modulated Fe solubility. Notably, soil 8 demonstrated measurable Cr(VI) retention despite alkaline conditions (pH 7.8), attributable to its exceptionally high

Exch-Fe(II) content (Table 4). This observation confirms Fe(II)‘s capacity to reduce Cr(VI) across wider pH ranges [18,37]. Fe(II) indeed serves as the primary reductant for Cr(VI) reduction,most of Cr(VI) tending to be reduced by Fe(II) in alkaline soil [18,38]. The concentration of Exch-Fe(II) seems a appropriate parameter to reflect the soil Fe species in reducing Cr(VI). Fe(II) can catalyze other reducing agents such as organic matter, sulfides, and microorganisms in Cr(VI) reduction through electron transfer processes, leading to the reducing agents more effective [39]. Specifically, Fe(II) reduces Cr(VI) to Cr(III), and the resulting Fe(III) is subsequently converted back to Fe(II) by the reducing agents [39].Thus, based on our findings, Exch-Fe(II) emerged as a key soil attribute influencing the retention of Cr(VI) and this result consistent with previous findings that soil Fe(II) plays a crucial role as a reductant in Cr(VI) retention [18,38,40].

Soil clay percentage is frequently correlated with Cr(VI) retention [13,14,16,41,42], however, this relationship is predominantly pH-mediated (indirect coefficient = 0.2655 vs. direct coefficient = 0.2265; Table 4). Combined with previous findings and our experimental results highlighting soil clay percentage emerged as an important parameter influencing Cr(VI) behavior only in acidic soils.Soil clay was an effective adsorbent and reducing agent for Cr(VI),especially in acidic condition [13,14]. Clay minerals always contain iron, either within their crystal structures or adsorbed/complexed on their surfaces which can facilitate adsorption on its surface followed by reduction by FeOx,and can release Fe(II) or Fe(III) to promote reduction with Cr(VI) under acidic conditions [13,14,41,42]. The significant correlation (*p < *0.05) between clay percentage and Exch-Fe(II), Amo-Fe, Com-Fe respectively (Table 3), along with the higher indirect coefficients of clay percentage with Exch-Fe(II) and Com-Fe (0.1556 and 0.1055, respectively, Table 4) further demonstrated that iron species in clay (especially Exch-Fe(II) and Com-Fe) maybe the important parameter determining Cr(VI) retention in soils. This hypothesis could be proven by other study of that Cr(VI) retention was significantly influenced by the concentration of structure Fe(II) in clay [42]. Thus Soil clay percentage should be interpreted cautiously as it was highly influenced by pH and irons pathway-both of which were the most important direct effective factors governing Cr(VI) retention (Fig 3).

### Effect of Com-Fe, CaCO_3_, and CEC

Com-Fe and CaCO3 exhibited context-dependent effects mediated through pH pathways (Table 4 and Fig 3).Com-Fe’s substantial indirect coefficient (0.4058, Table 4) suggests its primary influence occurs via pH-modulated Fe mobility rather than direct interaction. Com-Fe, which comprises organic substances containing functional groups such as phenolic hydroxyl and carboxyl groups, as well as bound Fe(II) and Fe(III), can readily form electron donors that accelerate Cr(VI) reduction under acidic conditionss [43,44]. Futhermore, Complex-Fe(II) can delay Fe(II) oxidation and maintain reducing ability in soils, which facilitated the reduction of Cr(VI) by OM [44]. However, due to the strong pH dependence and its influence on Cr(VI) retention via the Exch-Fe(II) and clay pathways (Fig 3), Com-Fe plays a less significant role compared to soil pH and exchangeable Fe(II). Contrasting soils 5 (pH = 5.72) and 6 (pH = 5.80) demonstrated that higher Com-Fe content (586.8 vs. 266.1 mg/kg) conferred no retention advantage under comparable pH conditions.

CaCO₃ content exhibited strong pH-dependent inhibitory effects on Cr(VI) reactions, as its indirect path coefficient (−0.2678) compared to the direct path coefficient (−0.1480) (Table 4).This reslut can be proved by the observation of that Group 4 (samples 9–16) which had roughly the same pH but significantly higher CaCO₃ levels than Group 3 (samples 9–12), resulting in negligible Cr(VI) reactivity. This phenomenon could be explained by two key mechanisms: (1) high CaCO₃ content reduces the availability of exchangeable sites for Cr(VI) adsorption, thereby lowering its adsorption capacity as CrO_4_^-^ or HCrO_4_^-^ competes with HCO_3_^-^ under pH 7–8 [45]; and (2) it promotes inherent Cr(VI) reduction and enhances Cr(VI) desorption [14]. Notably, other studies had reported consistent findings that saline-alkali soils (pH 7.0–8.5), characterized by high CaCO₃ content and extremely low Cr(VI) adsorption capacity, often exhibit a “trace reaction” phenomenon [22,45]. CaCO₃ content serves as a critical soil parameter influencing Cr(VI) retention under alkaline conditions, however, its impact becomes less pronounced across all pH ranges, as its effects were strongly dependent on pH.

CEC was smallest correlation coefficient with Cr(VI) retention (0.1049, Table 3), which seemed to have the least effect; however, it displayed a relatively higher direct correlation coefficient (−0.1251, Table 4). The relative independence of CEC appeared to exert a direct impact on Cr(VI) retention (Fig 3), whereas other studies have primarily reported correlations between CEC and Cr(VI) retention without emphasizing its direct effects [12,14,16]. CEC exhibited anomalous behavior in Soil 5 with lower pH and higher soil OM, where elevated CEC (49.35.cmol + /kg) coincided with unexpectedly low Cr(VI) retention, likely attributed to anion competition effects.Under tested soils (soil pH from 5.04 to 8.25), Cr(VI) primarily exists as anion of HCrO₄⁻ or CrO₄⁻ [36]. High CEC may enhances anion competition, reducing Cr(VI) adsorption potential and thereby decreasing Cr(VI) reduction since this reduction process depends on prior adsorption onto SOM and soil mineral surfaces [17,46,47].

### Effect of SOM, Er-Mn, and Amo-Fe

Numerous studies had reported a significant negative linear relationship between Cr(VI) retention capacity and SOM content [8,12–16,36]. In this study, although the correlation coefficient (*r* = 0.6515, *p* < 0.01, Table 1) was observed, the direct influence of OM on Cr(VI) retention was minimal (direct coefficient = 0.0472), whereas the indirect effect mediated by pH was substantial (indirect coefficient = −0.3030; Table 4). This challenges conventional assumptions regarding SOM’s direct role in Cr(VI) retention, instead demonstrating that SOM’s function is highly pH-dependent.For instance, contrasting soils 1 (pH = 5.04) and 12 (pH = 7.68) (Table 1)—which contained nearly equivalent SOM contents (3.6%)—revealed a complete divergence in Cr(VI) retention loss under acidic versus alkaline conditions (Table 2). Specifically, OM only facilitates Cr(VI) reduction under strongly acidic conditions. Dissolved organic matter (DOM) often regarded as the most active SOM exhibiting negligible Cr(VI) reduction at pH 6, and certain SOM types require even lower pH (pH < 4) [48,49]. Conversely, alkaline conditions completely inhibit both Cr(VI) adsorption and reduction by SOM [15,16].These studies indicate that soil pH is the primary determinant of the occurrence of Cr(VI)-SOM interactions and exerts the most critical control over these reactions. The significance of SOM in Cr(VI) retention should therefore be interpreted with reference to low pH conditions. Nevertheless, under the natural soil pH conditions examined in this study, SOM concentration emerged as a minor factor directly influencing Cr(VI) retention as overriding importance of soil pH.

However, it is not contradiction with the fact that adding OM to soil to remedy Cr(VI) is a better way to remedy Cr(VI) pollution soils, since adding OM mainly via indirectly ways in increasing Cr(VI) reduction such as proving better environment for reductant microbes [12,50], lower soil pH and increase soil reductants content,such as DOM and Fe(II) which could be reduced from soil clay-Fe(III) by some SOM [44].

Er-Mn content showed negligible direct effect (direct coefficient 0.0612, Table 4) with Mn influencing retention indirectly through Fe(II) availability (indirect coefficient −0.1578, Table 4).MnO_x_ are the mainly naturally occurring minerals capable of oxidizing Cr(III) to Cr(VI) [20,21,51]. Er-Mn, however, appears to had little direct effect on Cr(VI)-related reactions. This is because the oxidation of Cr(III) by Er-Mn is relatively weak and requires strict conditions, such as soils containing few other reductans and where Cr(III) remains unaged [51]. Additionally, the reduced Cr(VI) always exhibits resistance to reoxidation [51].Er-Mn may affect Cr(VI) retention by competing with reducing agents such as Fe(II), with a significantly larger indirect coefficient mediated by Fe(II) (Table 4 and Fig 3). Thus,soil Er-Mn content had little direct effect in Cr(VI) retention.

Amo-Fe also exhibited negligible direct effects on Cr(VI) retention, with its indirect influence mediated by pH (the indirect coefficient for pH was 0.1909, significantly larger than the direct coefficient of −0.032; Table 4). As the primary component governing soil adsorption capacity, Amo-Fe with huge specific surface area played a predominant role in adsorbing Cr(VI) [15,16,47]. The adsorption capacity of Amo-Fe for Cr(VI) is greatly affected by the pH environment [47]; thus, the absence of its anticipated direct role suggests its high pH dependence. Additional reasons for this result may include inhibition of its adsorption capacity by the background electrolyte (0.01 mol/L NO_3_^−^) [47], or predominance of reduction processes over adsorption in the tested soils [17].

### The soil properties affecting Cr(VI) retention by the soils in same pH

To investigate the decisive influence of soil pH and other key properties on Cr(VI) retention, this study adjusted soil soils to pH = 4.3, 6, and 8 using buffer solutions (Soil 16 exhibited unstable pH values at pH = 4.3 and 6 and was therefore excluded from these treatments). The results revealed a significant pH-dependent pattern. Specifically, Cr(VI) retention was highest at pH = 4.3, followed by a significant decrease at pH = 6 (71.60 ± 19.90%, Table 5) and 8 (88.69 ± 10.50%, Table 5). Notably, non-reactive soils exhibited reactivity toward Cr(VI) under acidic conditions (pH = 4.3), highlighting the critical role of pH in the regulation process (Table 5). Meanwhile, it is further supported that Exch-Fe(II) is a critical parameter for Cr(VI) retention, as soils containing higher Exch-Fe(II) (Soils 1–4 and 8) exhibited a smaller decrease in Cr(VI) retention at pH 6 and pH 8 compared to other soils.

**Table 5:** Cr(VI) retention in different soils under three pH treatments.

Soil number	Soil pH	Cr(VI) retention at nature pH	Cr(VI) retention in soils under different pH buffer		
			pH = 4.3 ± 0.1	pH = 6.0 ± 0.1	pH = 8.0 ± 0.1
1	5.04	198.95	207.15	120.33 (41.91%)	55.64 (73.14%)
2	5.28	179.62	178.63	89.13 (50.10%)	54.12 (69.70%)
3	5.34	161.97	158.03	84.10 (46.78%)	48.47 (69.33%)
4	5.38	155.20	145.75	61.96 (57.49%)	24.90 (82.92%)
5	5.72	57.31	76.44	8.62 (88.72%)	7.02 (90.82%)
6	5.80	64.51	98.23	28.24 (71.25%)	11.58 (88.21%)
7	6.14	50.77	83.97	23.21 (72.36%)	5.94 (92.93%)
8	7.55	42.85	141.79	88.12 (37.85%)	24.39 (82.80%)
9	7.27	11.19	81.99	7.60 (90.73%)	6.53 (92.04%)
10	7.60	9.29	70.10	22.20 (68.33%)	0.00 (100%)
11	7.44	6.83	105.75	12.14 (89.92%)	5.43 (94.87%)
12	7.68	6.27	173.88	43.34 (75.07%)	11.06 (93.64%)
13	7.37	0.00	61.39 (pH = 4.97)	4.59 (92.52%) (pH = 6.39)	0.00 (100%)
14	7.75	0.00	46.74 (pH = 4.70)	3.58 (92.34%) (pH = 6.48)	0.00 (100%)
15	7.84	0.00	38.42 (pH = 4.47)	0.00 (100%) (pH = 6.39)	0.00 (100%)

Note:The numbers in brackets are the percentage decrease in Cr(VI) retention at pH 6 or pH 8 relative to the retention measured at pH 4.3.

To mitigate multicollinearity between pH and other soil parameters, correlation analyses were recalculated under fixed soil pH conditions, as shown in Table 6. This approach enhances the accuracy of identifying key variables influencing Cr(VI) retention.When pH = 4.3, Exch-Fe(II) and SOM became extremely significant determinants (*p* < 0.01). At this soil pH, Com-Fe exhibited statistical significance (*p* < 0.05), while CaCO_3_, Er-Mn, and clay content showed a trend toward association.At pH = 6, Exch-Fe(II), Com-Fe, and clay content remained the primary contributing factors (*p* < 0.01).SOM and exchangeable manganese showed statistical significance (*p* < 0.05). CaCO_3_ and Amo-Fe exhibited potential influence. At pH = 8, the significance patterns shifted, with Exch-Fe(II), Com-Fe, clay, and SOM showing extremely significant effects (*p* < 0.01), Er-Mn maintaining moderate significance (*p* < 0.05), and CaCO_3_ and Amo-Fe again showing a trend toward association.

**Table 6:** Correlation coefficients of soil properties with Cr(VI) retention at pH 4.3, 6, 8.

Soil Properties	pH = 4.3	pH = 6	pH = 8
SOM (g/kg)	0.7911^**^	0.6388^*^	0.7185^**^
Exch-Fe(II)	0.73849^**^	0.9193^**^	0.8165^**^
Com-Fe	0.6808^*^	0.7243^**^	0.8280^**^
Clay	0.4771	0.7416^**^	0.7699^**^
Er-Mn	−0.5617	0.6503^*^	0.6540^*^
CaCO_3_	−0.5425	−0.45831	−0.4014
Amo-Fe	0.1557	0.3856	0.3613
CEC	−0.0183	−0.0531	−0.0583

Note:.“*” and “**” indicate significant (*p* < 0.05) and highly significant (*p* < 0.01) levels.

These findings generally align with the path analysis predictions, with Exch-Fe(II), Com-Fe, and clay identified as important factors in Cr(VI) retention under the same pH condition. However,unexpected result in the important role of SOM were oberserved. These results contradict the scientific hypothesis of this study, which posited that SOM plays a key role in the retention of Cr(VI) under relatively similar pH conditions. Soils 5 and 12, which had a higher SOM content, exhibits a lower Cr(VI) retention rate at natrual soil pH, supporting the path analysis conclusion that SOM is much less impotantance compared to soil pH.

However, this pattern exhibited contradictory changes in soil 5 (the black soil) at pH = 4.3. The SOM, clay, and Amo-Fe content of this sample were comparable to those of other samples, but the Cr(VI) retention was lower. This discrepancy suggests that the quality rather than the quantity of SOM may dominate the retention mechanism, which may be related to differences in functional groups or degree. Dissolved organic matter or undissolved humic acid (HA) tends to play a more important role in Cr(VI) retention [43,49,52]. Phenol and hydroxyl were determined as the main electron donors for Cr(VI) reduction at the black soil by HA and humin (HM), and Cr(VI) reduction was more dependent on aromatic carbon [52,53].Besides the type of organic matter and controlled by pH, the influence of soil minerals on organic matter is also significant.In soils enriched with crystalline FeO_x_, Cr(VI) reduction was primarily governed by SOM, while in soils enriched with poorly crystalline FeO_x_, mineral-associated Fe(II) contributed to Cr(VI) reduction [54]. The minerals in black soil dominated by montmorillonite and illite, had a low content of FeOx leading to relatively weak adsorption and reduction capacity [55].Thus, SOM-Minerals system should be considered together, as this mixed system’s effectiveness in Cr(VI) retention is affected by the types of SOM and soil minerals [40,42,43,56,57]. Soil 12 (Chernozem), compered to soil 5 (black soil), with the similar content of SOM,but much more crystalline form of FeOx, had higher Cr(VI) retention at pH 4.3. Crystalline FeO_x_ may be a important parameter for soil Cr(VI) retention as it can regulate the reaction of organic matter with Cr(VI).This also indicated that total SOM content was an unstable parameters for assess the Cr(VI) retention as it affected by SOM types, pH and soil minerals (crystalline FeO_x_).

Notably. The low Cr(VI) retention in soils 5−7 and 9−15 (pH = 4.3) may be attributed to higher Er-Mn and CaCO_3_ content, suggesting that Er-Mn and CaCO_3_ exert inhibitory effects through similar redox pathways. These findings suggest that Er-Mn may function similarly to CaCO_3_ in certain environments, participating in the immobilization process of Cr(VI).

## Conclusions

This study systematically elucidated the quantitative roles of key soil properties in governing Cr(VI) retention across 16 Chinese soils. Path analysis maybe a better way to analysis the mechanism of soil properties in affecting Cr(VI) retention than correlation analysis. Soil pH emerged as the dominant regulator, exhibiting the strongest direct negative effect on Cr(VI) retention (correlation coefficient = −0.9108, direct effect coefficient = −0.4867), with acidic soils (pH < 5.5) retaining 3−5 times more Cr(VI) than alkaline counterparts (pH > 7.5). Exch-Fe(II) served as the second critical factor (direct coefficient = 0.2324), directly reducing Cr(VI) at a wider range of soil pH and promoting the efficiency of SOM reduction of Cr(VI) under acidic soil (pH < 5.5). Clay content contributed significantly (direct coefficient = 0.2265), primarily through indirect pH-dependent mechanisms (indirect coefficient = 0.2655), enhancing Fe(II) availability from minerals. Com-Fe, despite its strong correlation with retention (*r* = 0.8856), exerted limited direct influence (direct coefficient = 0.1531), acting predominantly as a pH-modulated Fe reservoir. Conversely, CaCO_3_ inhibited retention (direct coefficient = −0.1480), particularly in alkaline soils, through pH elevation and competitive adsorption. SOM, Er-Mn, and Amo-Fe were found to exerted less direct influences on Cr(VI) retention (path coefficients<0.07) than other soil properties. Our analysis demonstratesthat SOM exhibits a significant pH-dependent in natural soil environments, whereby its becoming very important in Cr(VI) retention under conditions of relatively uniform pH condition and rich of Exch-Fe(II) or crystalline FeOx. Soil pH and Exch-Fe(II) are the most critical properties for assessing Cr(VI) rerention in soils, followed by the conten of Com-Fe and clay.Since Com-Fe and clay are Fe-bearing constituents, the species and abundance of other Fe-bearing substances (except Amo-Fe) may be key parameters governing this process.SOM content was an unstable parameters for assess the Cr(VI) retention in soils.

Furthermore, more soil geochemical parameters should be systematically integrated into field assessments to comprehensively characterize the multifaceted influences on Cr(VI) retention in natural soil systems,such as the types and content of Fe-bearing substances and SOM, soil texture, moisture regime, microbial community composition, and vegetation types. In the future, machine learning techniques applied to experimental and literature data may provide a superior method for evaluating the site-specific environmental behavior of chromium Cr(VI) in soil.

## Supporting information

S1 File Soil Types Characteristics. (DOC)

S2 File The methods for soil characteristics measurement. (DOC)

S1 Images S1 raw images. (PDF)

S3 File S3 Calculation Method of Path Analysis. (DOC)
