# Supplementary material for: Key soil properties governing Cr(VI) retention in 16 natural soils: A comprehensive geochemical and statistical analysis
Source: PLoS One. 2025 Dec 22;20(12):e0338375. doi: 10.1371/journal.pone.0338375 (PMC12721535; doi:10.1371/journal.pone.0338375)
Supplement: S1 File — (DOC) [file pone.0338375.s001.doc]

**Soil Type Characteristics**

Chinese Soil Classification was based on soil genesis.A systematic classification of Chinese soils has been accomplished by integrating soil-forming conditions,soil-forming processes, and soil properties (including morphological, physical, and chemical properties).

| **Sample** | **Soil Types** | **Characteristics** | **Physicochemical properties** |
| --- | --- | --- | --- |
| 1 | Yellow Soil | Belongs to the Alfisols group, mainly derived from the weathering of acidic rock parent materials such as granite and sandstone. Under the subtropical high-humidity environment, weathering is moderate and eluviation is relatively strong; iron oxides in the soil are mainly in hydrated form ( yellow color). | pH value: 4.5-5.5 (strongly acidic);  Organic matter content: 1%-3% (over 5% in forested areas);  Clay content: 20%-40%;  CEC: 5-15 cmol (+)/kg (low);  Iron oxide content: moderate, mainly hydrated iron oxides. |
| 2 and 3 | Red Soil | Belongs to the Alfisols group, derived from the intense weathering of parent materials such as granite, gneiss, and sandstone. Under the subtropical high-temperature and high-humidity environment, weathering and eluviation are intense, with significant accumulation of iron oxides in the soil (red color). | pH value: 4.0-5.5 (strongly acidic);  Organic matter content: 1%-2% (easily drops below 1% after reclamation);  Clay content: 30%-50% (high);  CEC: 5-10 cmol (+)/kg (extremely low); Iron oxide content: high, mainly hematite and goethite. |
| 4 | Lateritic Red Soil | Belongs to the Alfisols group (transitional type between Red Earth and Latosol), derived from the weathering of acidic rock parent materials. Under the south subtropical hot and humid environment, weathering and eluviation are stronger than those of Red Earth, with more obvious accumulation of iron and aluminum. | pH value: 4.0-5.0 (strongly acidic);  Organic matter content: 1%-2.5%;  Clay content: 35%-55% (extremely high); CEC: 4-12 cmol (+)/kg (low);  Iron oxide content: extremely high, with a high proportion of hematite. |
| 5 | Black Soil | Belongs to the Mollisols group, developed from loess-like sediment parent materials. Under the temperate cool and humid environment, organic matter accumulates vigorously (slow decomposition of steppe meadow vegetation residues), with weak weathering and eluviation. | H value: 5.5-7.0 (neutral to slightly acidic); Organic matter content: 5%-10% (over 15% in some areas, extremely high);  Clay content: 20%-30% (moderate);  CEC: 20-40 cmol (+)/kg (extremely high); Iron oxide content: low, mainly in weakly crystalline form. |
| 6 | Yellow-  Brown Soil | Belongs to the Alfisols group (transitional type between Yellow Earth and Brown Earth), derived from the weathering of various parent materials (e.g., sandstone, granite). Under the northern subtropical mild and humid environment, weathering and eluviation are moderate, with weak clayification. | pH value: 5.0-6.5 (acidic to slightly acidic); Organic matter content: 2%-4%;  Clay content: 20%-35% (moderate);  CEC: 10-20 cmol (+)/kg (moderate);  Iron oxide content: moderate, with low crystallinity. |
| 7 | Yellow-  Cinnamon Soil | Belongs to the Alfisols group, mainly derived from the weathering of Quaternary red clay parent materials. Under the northern subtropical humid environment, eluviation is relatively strong, with significant clayification (forming a thick clay-enriched layer). | pH value: 5.5-7.0 (slightly acidic to neutral);  Organic matter content: 1%-2.5%;  Clay content: 40%-60% (extremely high); CEC: 15-25 cmol (+)/kg (moderate);  Iron oxide content: relatively high, often forming iron-manganese concretions.​ |
| 8 | Paddy Soil | Belongs to the Anthrosols group, transformed from natural soils (e.g., Red Earth, Yellow Earth, Fluvo-aquic Soil) through long-term paddy cultivation (alternating dry and wet conditions). Parent materials are diverse, and the formation process is dominated by human farming and alternating redox reactions. | H value: 5.5-7.5 (slightly acidic to neutral in southern China);  Organic matter content: 2%-4% (over 5% in high-fertility plots);  Clay content: 15%-45% (varies with parent materials);  CEC: 10-25 cmol (+)/kg (moderate);  Iron oxides show alternating redox states, with iron-manganese mottles formed in some parts. |
| 9 | Fluvo-  Aquic Soil | Belongs to the Inceptisols (semi-hydromorphic soils) group, developed from fluvial alluvial parent materials. Influenced by the seasonal fluctuation of groundwater, weathering is weak, the soil retains alluvial stratification, and eluviation is moderate. | pH value: 6.5-8.5 (neutral to slightly acidic in southern China,);  Organic matter content: 1%-3%;  Clay content: 10%-40% (large variation); CEC: 15-25 cmol (+)/kg (moderate);  Iron oxide content: moderate, evenly distributed. |
| 10 | Cinnamon Soil | Belongs to the Alfisols group, derived from the weathering of loess parent materials. Under the warm temperate semi-humid environment, weathering is moderate, eluviation is weak, and some plots have a caliche layer. | pH value: 7.0-8.5 (neutral to slightly alkaline);  Organic matter content: 1%-2.5%;  Clay content: 20%-35% (moderate);  CEC: 15-25 cmol (+)/kg (moderate);  Iron oxide content: low, with high crystallinity. |
| 11 | Castanozems | Belongs to the Aridisols group, derived from the weathering of loess-like sediments and residual parent materials. Under the temperate semi-arid environment, weathering and eluviation are weak, with significant calcification (forming a caliche layer below the surface). | pH value: 7.5-9.0 (slightly alkaline to alkaline);  Organic matter content: 1%-2%;  Clay content: 15%-25% (relatively low); CEC: 10-20 cmol (+)/kg (low);  Iron oxide content: low, mostly coated by calcium carbonate. |
| 12 | Chernozem | Belongs to the Mollisols group, developed from loess-like sediment parent materials. Under the temperate semi-humid grassland environment, organic matter accumulates richly (meadow steppe vegetation), eluviation is weaker than that of Black Soil, with a deep caliche layer. | pH value: 6.5-8.0 (neutral to slightly alkaline);  Organic matter content: 3%-6% (high); Clay content: 15%-25 % (moderate);  CEC: 20-40 cmol (+)/kg (high);  Iron oxide content: predominantly in crystalline forms. |
| 13 | Cultivated Loessial soils | Belongs to the Inceptisols (primitive soils) group, directly developed from loess parent materials (low weathering degree, short soil-forming time). Under the temperate arid and windy environment, the structure is loose, prone to erosion, with no obvious eluviation or clayification. | pH value: 7.5-8.5 (slightly alkaline to alkaline);  Organic matter content: 0.5%-1.5% (extremely low);  Clay content: 10%-20% (low);  CEC: 5-15 cmol (+)/kg (extremely low); Iron oxide content: low, evenly distributed. |
| 14 | Lou Soil | Belongs to the Anthrosols group, formed from loess parent materials through long-term cultivation and stacking maturation. The surface stacked layer is thick, and soil fertility is enhanced by artificial fertilization and cultivation, with moderate weathering. | pH value: 7.0-8.0 (neutral to slightly alkaline);  Organic matter content: 1.5%-3%;  Clay content: 20%-30% (moderate);  CEC: 18-25 cmol (+)/kg (moderate to slightly high);  Iron oxide content: low, with moderate crystallinity. |
| 15 | Purple Soil | Belongs to the Inceptisols (primitive soils) group, rapidly derived from the weathering of purple sandstone parent materials (fast weathering speed, short soil-forming time). Under the subtropical environment, eluviation is weak, and the soil retains the purple color and mineral composition of the parent material. | pH value: 5.5-8.0 (acidic to slightly alkaline, fluctuating with parent materials); Organic matter content: 1%-2.5%;  Clay content: 15%-40% (large variation); CEC: 10-20 cmol (+)/kg (moderate);  Iron oxide content: moderate, showing purple color due to superposition with parent material color. |
| 16 | Cumulated Irrigated soils | Belongs to the Anthrosols group, formed by long-term irrigation silting (silted layer thickness > 50 cm) of fluvial sediments and cultivation improvement. Under the arid and semi-arid environment, eluviation is weak, and some areas have salt accumulation. | pH value: 7.5-9.5 (alkaline to strongly alkaline);  Organic matter content: 1%-3% (greatly affected by fertilization);  Clay content: 20%-35% ;  CEC: 15-25 cmol (+)/kg (moderate);  Iron oxide content: low, evenly distributed. |
