# Supplementary material for: Key soil properties governing Cr(VI) retention in 16 natural soils: A comprehensive geochemical and statistical analysis
Source: PLoS One. 2025 Dec 22;20(12):e0338375. doi: 10.1371/journal.pone.0338375 (PMC12721535; doi:10.1371/journal.pone.0338375)
Supplement: S2 File — (DOC) [file pone.0338375.s002.doc]

**The methods for soil characteristics measurement**

**1.PH Measurement**:Soil samples with deionized water(solid-liquid mass ratio 1:1) that has had CO_2_ removed(prepared by boiling distilled water for 10 min to expel dissolved CO₂, then cooled to 25°C), were shaked at 180 rpm for 1 hour ,and then allowed to stand overnight at 25±1°C to equilibrate, and a pH meter (PHS-3C model, Inesa scientific instrument Co., Ltd., China) was used to determine the pH of the soil solution.

**2.SOM Measurement**:SOM was determined via the modified Walkley-Black method (K_2_Cr_2_O_7_-H_2_SO_4_ oxidation), which oxidizes organic carbon (SOC) and converts results to SOM using a stoichiometric coefficient(SOM=SOC×1.724). 0.5 g (±0.0001 g) soil into a flask, add 5 mL 0.8 mol/LK_2_Cr_2_O_7_-H_2_SO_4_ and 5 mL concentrated H_2_SO_4_, then heat at 170–180°C for 5–10 min (until greenish-yellow). Cool, dilute to ~150 mL with deionized water.Add 10 drops of o-phenanthroline indicator, titrate with standardized (NH_4_)_2_Fe(SO_4_)_2_ until color shifts from orange-red to green.

**3.Soil Clay Content Measurement**: Soil was treated with H_2_O_2_(H_2_O_2_ : water = 1:4) to remove OM, followed by particle size distribution analysis using a [Laser](http://dict.cnki.net/dict_result.aspx?searchword=激光粒度仪&tjType=sentence&style=&t=laser+particle+size+analyzer) [particle size analyzer](http://dict.cnki.net/dict_result.aspx?searchword=粒度仪&tjType=sentence&style=&t=particle+size+analyzer) (Malvern MASTERSIZER 3000 model, UK), and data processing[23].

**4.Soil Exch-Fe(II) Concentration Measurement**:soils were extracted with 0.1 mol/L Al_2_(SO_4_)_3_, and detected Fe(II) in extracts by the spectrometer using the phenanthroline method.2.000 g (±0.001 g) soil was mixed with 25 mL 0.1 mol/L Al_2_(SO_4_)_3_ (25°C) in a 50 mL polyethylene tube, shaken horizontally at 180 rpm for 30 min, then centrifuged at 3000 rpm for 15 min. 10 mL of the supernatant was transferred to a new tube, treated with 1 mL 10% NH_2_OH·HCl , mixed with 2 mL acetate buffer (pH 5.5) and 1 mL 0.1% phenanthroline, shaken, and incubated in the dark at 25°C for 15 min. Fe(II)-phenanthroline complex absorbance at 510 nm was measured (blank with deionized water). Exch-Fe(II) concentration was calculated using a standard curve and the formula:Exch-Fe(II)=C_extract_×0.01×1000/2000​, where C_extract_(mg/L) is the extract Fe(II) concentration[23].

**5.Soil Amorphous Fe Oxides(Amo-FeO_x_) content Measurement**: Soils were extracted by 0.2 mol/L (NH_4_)_2_C_2_O_4_, and detected total iron in extracts by Atomic Absorption Spectroscopy (AAS, iCE 3500 model, Thermo Fisher Scientific, USA)[23].2.000 g (±0.001 g) of soil was transferred to a 50 mL polyethylene tube, mixed with 25 mL of 0.2 mol/L (NH₄)₂C₂O₄ solution, sealed, and horizontally shaken at 180 rpm for 30 min to dissolve Amo-FeOx. After centrifugation at 3000 rpm for 15 min, 20 mL of the supernatant was collected for total Fe analysis. Fe concentration in the extract was determined via a AAS spectrometer at 248.3 nm. Amo-FeOx content (g/kg) was calculated using the formula:Amo-FeOx=C_extract_×V_extract_×D/m,where C_extract_(mg/L) is the Fe concentration in the extract,V_extract_(L) is the extract volume (0.02 L for 20 mL),D is the dilution factor (1 if no dilution), and m(kg) is the dry soil weight 0.002 kg.

**6.Soil Complexed-Fe(Com-Fe) content Measurement**: the soils were extracted by 0.1 mol/L,Na₄P₂O₇ (pH=8.5), and detected iron in extracts by AAS[23]. **Determination of Soil Com-Fe Content**. 2.000 g (±0.001 g) of soil was transferred to a 50 mL polyethylene tube, mixed with 25 mL of the 0.1 mol/L Na₄P₂O₇ solution (pH 8.5), sealed, and horizontally shaken at 180 rpm for 30 min. After centrifugation at 3000 rpm for 15 min, 10 mL of the supernatant (avoiding pellet disturbance to prevent Fe re-adsorption) was collected for analysis. Fe concentration in the extract was measured via a AAS spectrometer at 248.3 nm,. Com-Fe content (mg/kg) was calculated using the formula: Com-Fe=C_extract_×V_extract_×D/m, where C_extract_(mg/L) is the Fe concentration in the extract,V_extract_(L) is the extract volume (0.01 L for 10 mL),D is the dilution factor (1 if no dilution), andm(kg) is the dry soil weight 0.002 kg.

**7.Soil Easily Reducible Manganese Content(Er-Mn) Measurement**: soil was extracted by 1.0 mol/L,MgSO_4_ and 1.0 mol/L MgSO_4_-0.2 M hydroquinone (1:1) respectively, measured the total manganese by AAS, the concentration of Er-Mn were the manganese difference between these two extracts [22-23].2.000 g ± 0.001 g Soil was subjected to two extractions: (1) blank extraction with 25 mL 1.0 mol/L MgSO₄ (shaken at 180 rpm for 30 min, 25°C) to isolate non-reducible Mn; (2) reducing extraction with 25 mL 1.0 mol/L MgSO₄-0.2 mol/L hydroquinone (1:1 v/v, same shaking conditions) to dissolve Ered-Mn as soluble Mn²⁺. After centrifugation (3000 rpm, 15 min), 10 mL supernatant from each extraction was collected . Mn concentrations in extracts were measured via a AAS spectrometer at 279.5 nm. Ered-Mn content (mg/kg) was calculated as the difference between reducing and blank extracts using:Er-Mn=(Crecuing-Cblank)×Vextract×D/m,where C_reducing_ and C_blank_(mg/L) are Mn concentrations in reducing/blank extracts,V_extract_ is extract volume,D is the dilution factor (1 if no dilution), and m=0.002 kg.

**8.Soil CaCO_3_ content:** It was measured by the [gasometric method](http://dict.cnki.net/dict_result.aspx?searchword=简易气量法&tjType=sentence&style=&t=facility+gasometric+method) by measuring CO_2_ gas released from the reaction of CaCO_3_ with excess hydrochloric acid (HCl).Fill the gas burette with CO₂-free water, invert it into a water bath, and record the initial volume (V_0_). Allow the system to equilibrate to 20°C for 15 min.Weigh 0.5 g (±0.0001 g) of air-dried soil into the reaction flask.Add 25 mL of standardized 0.2 mol/L HCl to the flask using a pipette. Quickly seal the flask with the stopper, ensuring no air bubbles are trapped in the connecting tube.Swirl the flask gently for 10–15 s to initiate reaction, then place it in a 20°C water bath. Allow the reaction to complete(60 Min) After reaction completion, adjust the burette height to equalize pressure inside the system. Record the final gas volume (V_1_)，Correct the measured CO_2_ volume (V_1_–V_0_–V_blank_) to standard temperature and pressure using the ideal gas law.Soil CaCO_3_ content (g/kg) was calculated using the stoichiometry of the reaction.

**9.Cation Exchange Capacity (CEC) Measurement**: CEC was determined by the method of ammonium acetate exchange. 2.000 g (±0.001 g) of soil was transferred to a 50 mL polyethylene tube, mixed with 25 mL of 0.1 mol/L NH₄OAc solution (pH 7.0, adjusted using 1 mol/L NH₄OH or HNO₃), sealed tightly, and shaken horizontally at 180 rpm for 60 min at 25°C to maximize displacement of exchangeable cations.After shaking, the mixture was centrifuged at 3000 rpm for 15 min to separate the supernatant. The supernatant was decanted and discarded. Fresh 0.1 mol/L NH₄OAc solution (25 mL) was added to the soil pellet, and the shaking-centrifugation cycle was repeated twice more to ensure complete cation exchange. Following the third extraction, the supernatant was discarded, and the soil pellet was washed with 25 mL of 95% ethanol three times.For NH₄⁺ displacement and quantification, the ethanol-washed soil pellet was transferred to a 100 mL conical flask, mixed with 50 mL of 1 mol/L KCl solution, sealed, and shaken vigorously for 30 min at 25°C. The mixture was then centrifuged at 3000 rpm for 15 min, and the supernatant was collected and filtered through a 0.45 μm cellulose acetate membrane to remove particulates. NH₄⁺ concentration in the filtrate was determined using a colorimetric method. A reagent blank (25 mL NH₄OAc solution + 25 mL KCl solution, processed identically to samples) was analyzed to correct for background NH₄⁺ contamination.Cation exchange capacity (cmol⁺/kg) was calculated using the formula:CEC=(C_sample_-C_blank_)×V_KCl_×10/m，where C_sample_ is concentration in the sample extract (mmol/L),C_blan_k is NH₄⁺ concentration in the blank extract (mmol/L),VKCl is Volume of KCl solution (L; 0.05 L for 50 mL),m is dry weight of soil sample 0.002 kg,10: Conversion factor from mmol to cmol (1 cmol = 10 mmol).
