# Supplementary figures and images for: Key soil properties governing Cr(VI) retention in 16 natural soils: A comprehensive geochemical and statistical analysis
Source: PLoS One. 2025 Dec 22;20(12):e0338375. doi: 10.1371/journal.pone.0338375 (PMC12721535; doi:10.1371/journal.pone.0338375)

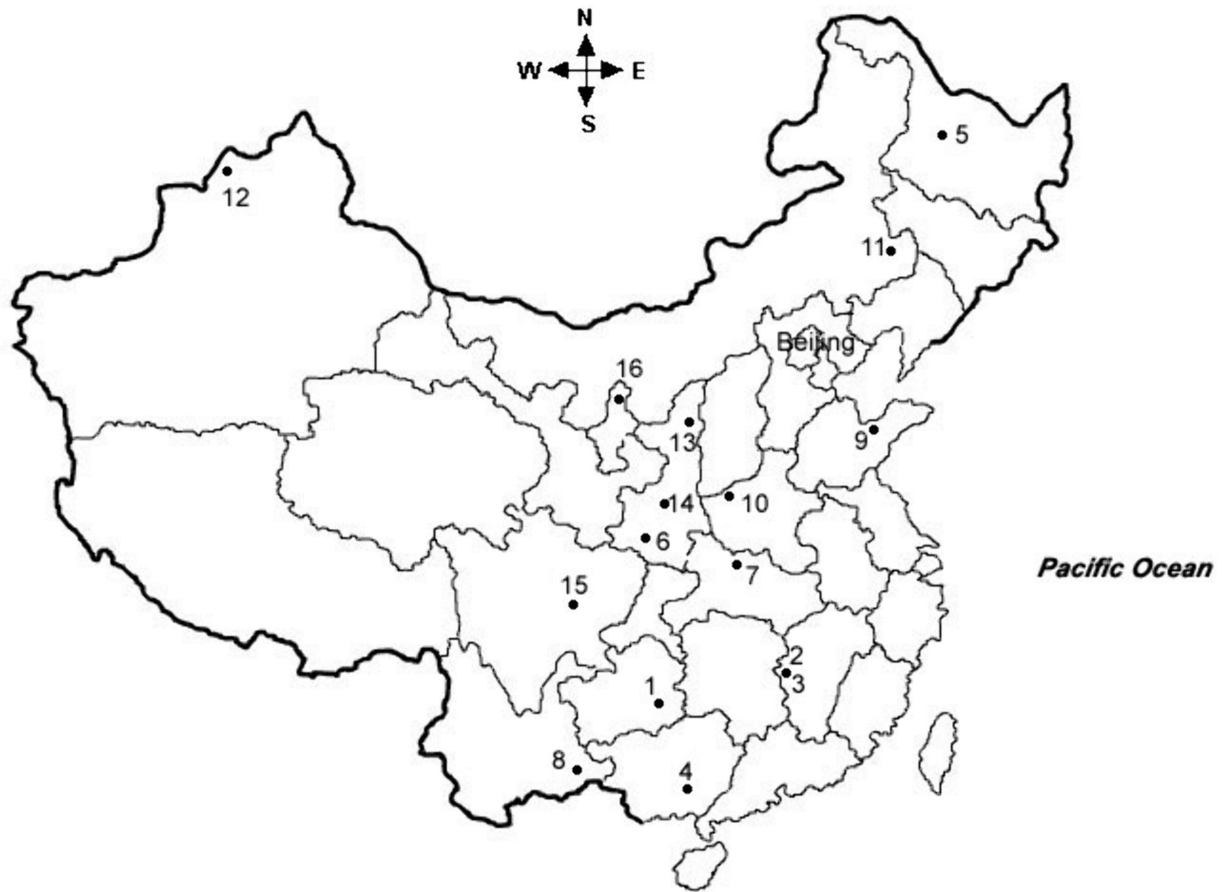

Figure1.tif

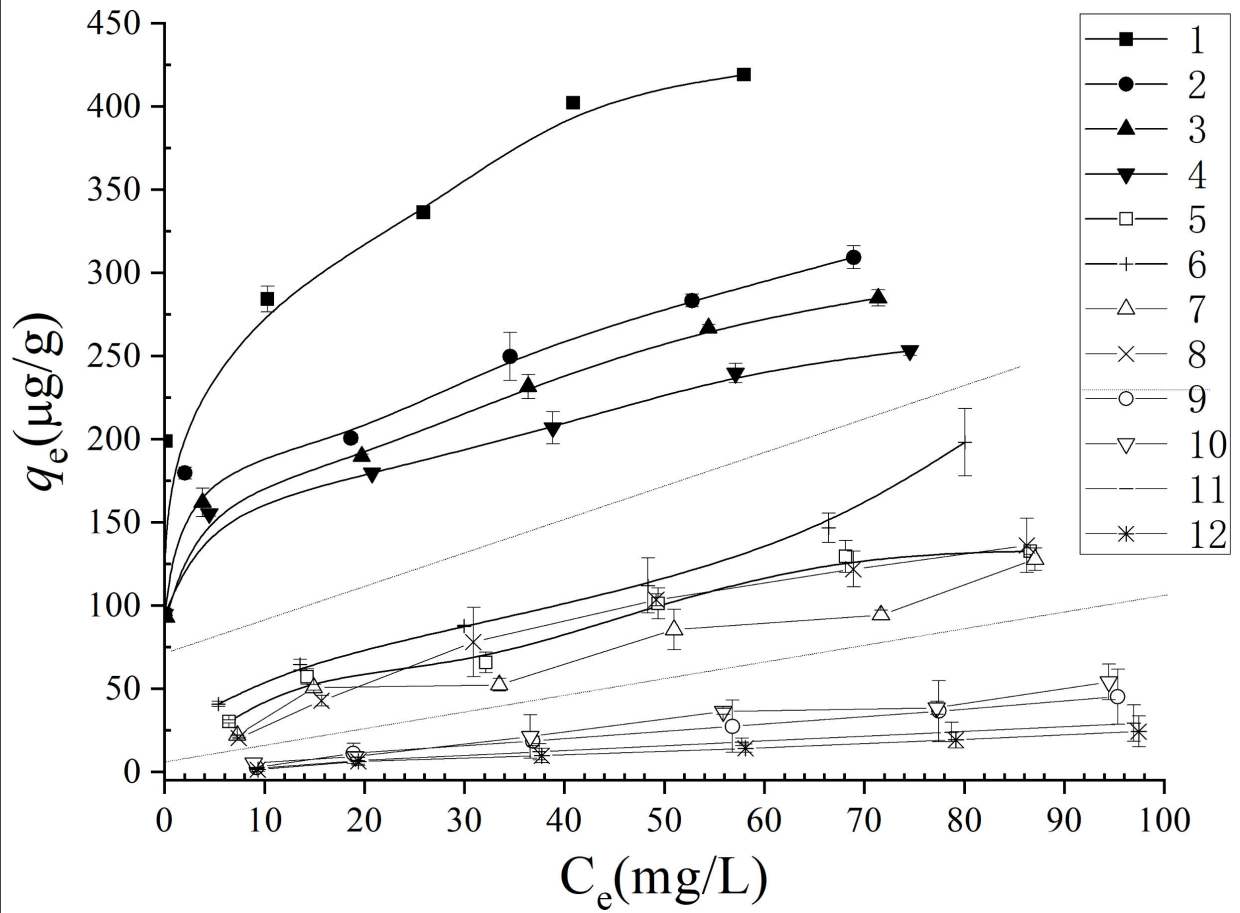

Figure2.tif

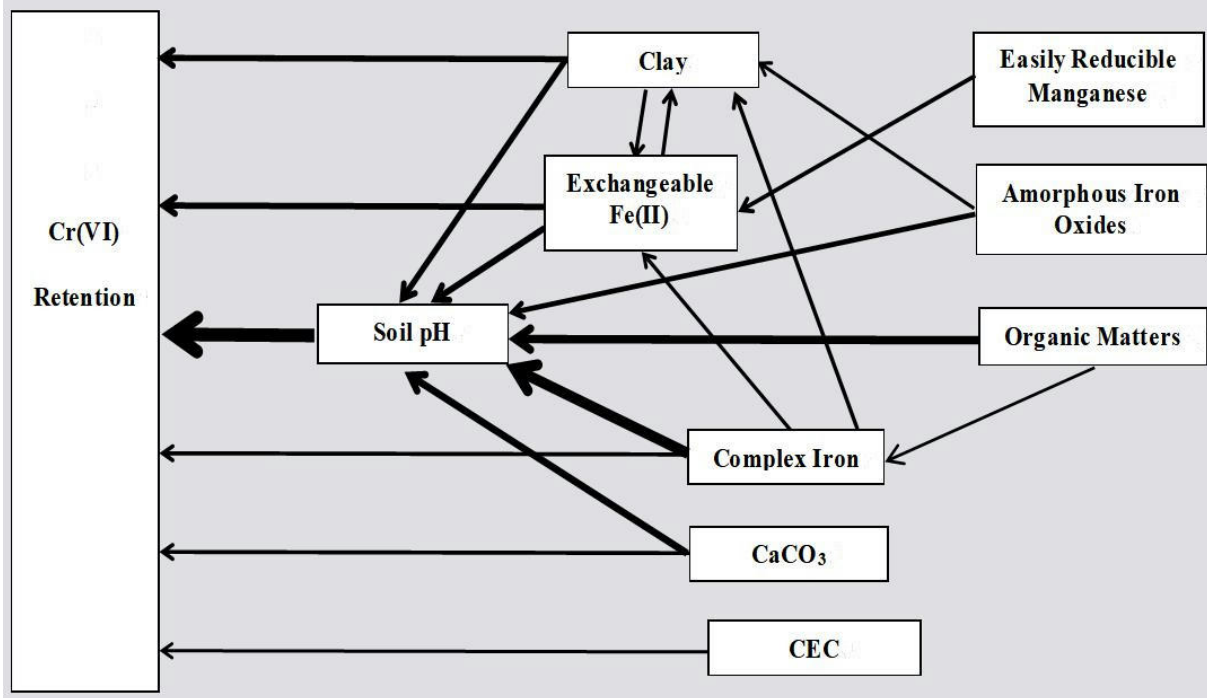

Figure3.tif

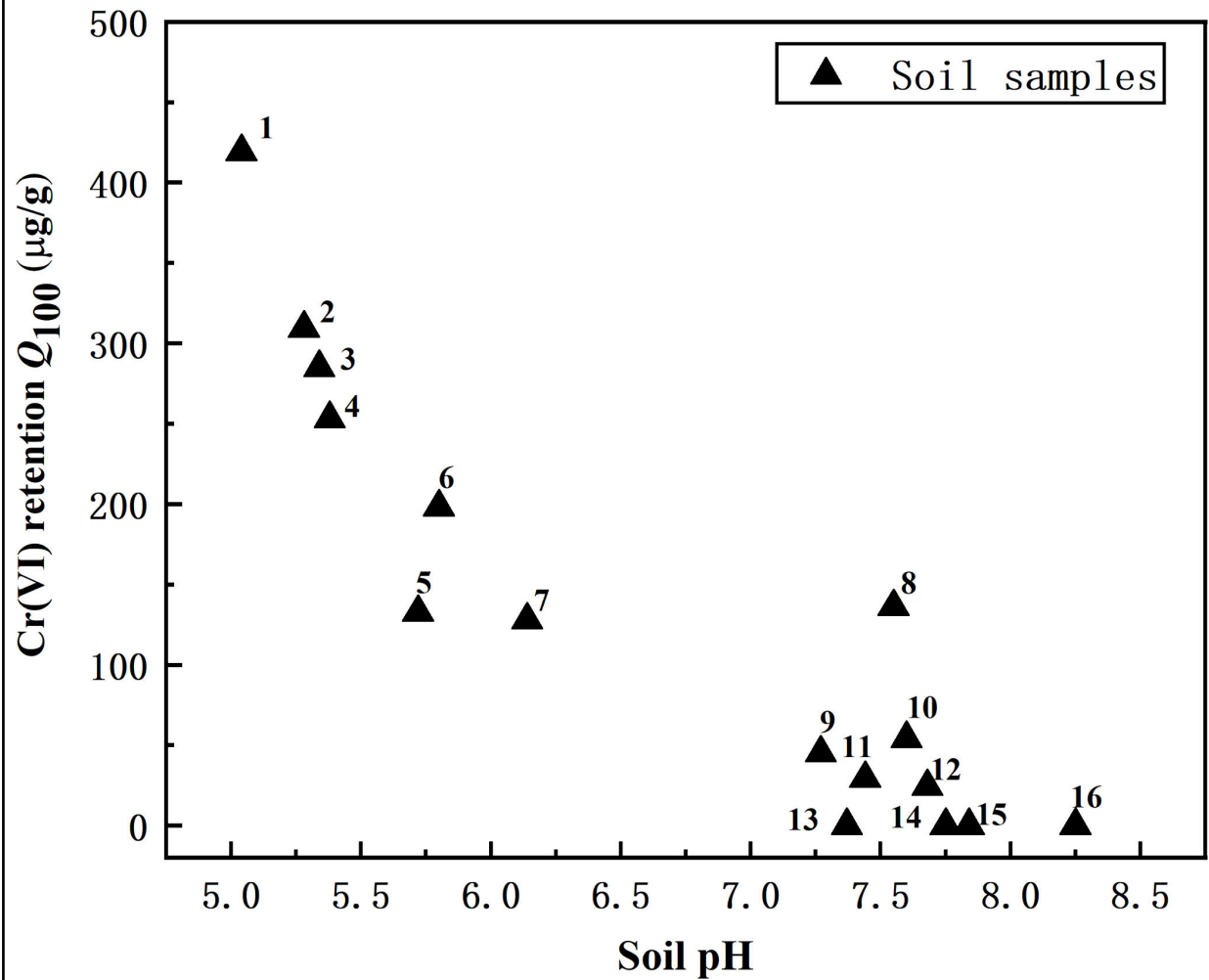

Figure4.tif

Supplement: S1 Images — (PDF) [file pone.0338375.s003.pdf]
