# Supplementary material for: Key soil properties governing Cr(VI) retention in 16 natural soils: A comprehensive geochemical and statistical analysis
Source: PLoS One. 2025 Dec 22;20(12):e0338375. doi: 10.1371/journal.pone.0338375 (PMC12721535; doi:10.1371/journal.pone.0338375)
Supplement: S3 File — (DOC) [file pone.0338375.s004.doc]

# **Calculation Method of Path Analysis**

## **1 Analysis Overview**

### **1.1 Analysis Purpose**

Based on the correlation coefficient matrix of soil properties and Q_100_, decompose the direct effects, indirect effects, and contribution degrees of each soil property on Q_100_ through path analysis to identify key influencing factors.

### **1.2 Variable System**

- •****Target variable****: Q_100_.
- •****Explanatory variables****: 9 soil properties: pH, SOM, Exch-Fe(II), Amo-Fe, Com-Fe, Er-Mn, CEC, Clay, CaCO₃.

### **1.3 Analysis Method**

Path Analysis, with core formulas including:

- •Direct path coefficient:*P*=*R*−1×*r*
- •Total indirect effect: Indirect effect=∑(*rij*×*Pj*)(*j*=*i*)
- •Coefficient of determination: Direct coefficient of determination=*P*2, Indirect coefficient of determination=2×*rij*×*Pi*×*Pj*, Total coefficient of determination*R*2=∑(direct coefficients)+∑(indirect coefficients)

## **2 Basic Data**

### **2.1 Correlation Coefficient Matrix**

Based on the Table 3, extract the complete correlation coefficient matrix (10×10), covering all pairwise correlations between the 9 explanatory variables and the target variable Q_100_.

### **2.2 Core Matrix Definitions**

#### **2.2.1 R Matrix (Correlation Matrix Among Explanatory Variables)**

A 9×9 matrix where element *R*[*i*][*j*]represents the correlation coefficient between the i-th and j-th explanatory variables.

#### **2.2.2 r Vector (Vector Correlated with Q100)**

A 9×1 vector where element *r*[*i*]represents the total correlation coefficient between the i-th explanatory variable and Q_100_.

## **3 Calculation Process**

### **3.1 Step 1: Extract Core Matrices**

Extract the R matrix (9×9) and r vector (9×1) from the complete correlation coefficient matrix as follows:****R Matrix (first 3×3 example)****:

| **Explanatory Variable** | **pH** | **SOM** | **Exch-Fe(II)** |
| --- | --- | --- | --- |
| pH | 1.0000 | -0.6226 | -0.4884 |
| SOM | -0.6226 | 1.0000 | 0.4239 |
| Exch-Fe(II) | -0.4884 | 0.4239 | 1.0000 |

****r Vector (complete data)****:

| **Explanatory Variable** | **Correlation Coefficient with Q100 (*rxy*)** |
| --- | --- |
| pH | -0.9108 |
| SOM | 0.6515 |
| Exch-Fe(II) | 0.7487 |
| Amo-Fe | 0.4289 |
| Com-Fe | 0.8856 |
| Er-Mn | -0.3910 |
| CEC | 0.1049 |
| Clay | 0.7146 |
| CaCO₃ | -0.5169 |

### **3.2 Step 2: Calculate Inverse Matrix of R (*R*−1)**

Obtain *R*−1(9×9) through matrix inversion, satisfying *R*×*R*−1=*I*(identity matrix), and verify the correctness of matrix inversion .****Inverse Matrix of R (first 3×3 example)****:

| **Explanatory Variable** | **pH** | **SOM** | **Exch-Fe(II)** |
| --- | --- | --- | --- |
| pH | 6.2143 | -0.6614 | -1.4978 |
| SOM | -0.6614 | 3.4961 | -1.1429 |
| Exch-Fe(II) | -1.4978 | -1.1429 | 8.5148 |

### **3.3 Step 3: Calculate Direct Path Coefficients (P)**

Calculate direct path coefficients according to the formula *P*=*R*−1×*r*(matrix multiplication), with results as follows:

| **Rank** | **Explanatory Variable** | **Direct Path Coefficient (P)** |
| --- | --- | --- |
| 1 | pH | -0.4867 |
| 2 | Exch-Fe(II) | 0.2324 |
| 3 | Clay | 0.2265 |
| 4 | Com-Fe | 0.1531 |
| 5 | CaCO₃ | -0.1480 |
| 6 | CEC | -0.1251 |
| 7 | Er-Mn | 0.0612 |
| 8 | SOM | 0.0472 |
| 9 | Amo-Fe | -0.0332 |

*Calculation basis: P=R−1×r, matrix multiplication.*

The indirect contribution of interactions among variables to Q_100_, with the formula: Indirect coefficient of determination = 2×r_ij×P_i×P_j​ (where i<j to avoid duplicate calculations).
